# Spatiotemporal Changes of Cultivated Land System Health Based on PSR-VOR Model—A Case Study of the Two Lake Plains, China

**DOI:** 10.3390/ijerph20021629

**Published:** 2023-01-16

**Authors:** Xigui Li, Qing Wu, Yujie Liu

**Affiliations:** 1 College of Landscape Architecture and Art Design, Hunan Agricultural University, Changsha 410128, China; 2 Tourism and Historical Culture College, Zhaoqing University, Zhaoqing 526061, China; 3 School of Geography, Nanjing Normal University, Nanjing 210023, China; 4 The College of Urban & Environmental Sciences, Central China Normal University, Wuhan 430079, China

**Keywords:** cultivated land system health, PSR-VOR model, zoning regulation, two lake plains

## Abstract

Cultivated land resources are the material basis of sustainable agricultural development. Climate change, food security, land pollution, and other issues highlight the value of sustainable agricultural development, and the health of the cultivated land system has attracted much attention. By constructing “PSR-VOR” cultivated land system health evaluation framework under the 5 km grid scale and using GIS spatial analysis and mathematical statistics to comprehensively evaluate the health status of the cultivated land system in the two lake plains from 2000 to 2019. The major results have shown that: (1) Over the past 20 years, both the highest and average values of the health index of the cultivated land system have gone down, and the health status of the cultivated land system has changed and gotten worse over time. (2) The health status in the two lake plains has been generally good, mainly in Class I and Class II areas. However, the area of cultivated land with general and poor health status has increased rapidly. On the whole, the health level presents the characteristic of gradually decreasing from the northeast to the southwest and southeast. (3) During the study period, the global Moran’s I value of the cultivated land system health index in the two lake plains increased from 0.686 to 0.729, with significant spatial positive autocorrelation, and the spatial heterogeneity of the cultivated land system health index gradually increased. As shown by the spatial distribution characteristics of high in the north, low in the south, and decreasing from the middle to the outside, the distribution of the high-value cluster area and the low-value cluster area of the cultivated land system health index in the two lake plains has not changed significantly over the past 20 years. (4) The two lake plains are divided into five areas: a moderate optimization area, a collaborative optimization area, a potential improvement area, a key improvement area, and a priority improvement area. The urgency of regulating the health status from the moderate optimization area to the priority improvement area has gradually increased, and the differentiated utilization and management of cultivated land resources need to be carried out according to local conditions.

## 1. Introduction

As the essence of land resources, cultivated land is not only the basic material for human beings to engage in agricultural production activities, but also the important material basis for achieving sustainable social and economic development and maintaining national food security [1]. The way that farmed land is used is significantly influenced by human activity. The reclamation of cultivated land resources, the change of planting structure, and the abandonment of cultivated land will change the energy flow, information flow, and material flow of cultivated land, thus affecting the global change process of climate, biology, hydrology, and so on in the land surface system [2,3]. China’s industrialization and urbanization have advanced since the 1980s, causing an unprecedented transformation of the cultivated land resources and the emergence of issues like the disorganized growth of construction land, the dramatic reduction of cultivated land resources, and the degradation of the quality of cultivated land [4,5]. Human activities have a significant negative influence on the structure and function of the cultivated land system, the contradiction between human and land is becoming increasingly acute, and the health of the cultivated land system is deteriorating gradually, which endangers national food security, agricultural modernization and the sustainable development of the social economy [6]. To guarantee the continuing and sustainable growth of the regional agricultural sector, it is essential to analyze the overall health of the cultivated land system and to manage the interactions between food security, economic development, and ecological security.

In the 1940s, Aldo Leopold, who believed that the health of land refers to the situation after being occupied and used by human beings, but that the function is not destroyed, put forward the academic concept of “land health” and defined its connotation [7], regarding “land organism health” as an important ability of self-renewal within the system [8]. On this basis, the academic community has carried out research on ecosystem health, land use health, agricultural ecosystem health, soil health, and other aspects [9,10,11], with an emphasis on the effect of land use change on the health status of vegetation [12], soil [13], hydrology [14], and cultivated land [15]. The advancement of agricultural intensification, irrational fertilization, farming, grazing, crop types, and crop rotation plans will have a significant impact on the health of the cultivated land system [16], and environmental issues such as soil erosion, pollution, and biodiversity loss will become more prevalent. Agriculture has become the leading cause of environmental degradation on a global scale [17,18]. People have started to become more aware of the intimate connection that exists between healthy land and healthy people as a result of the complete promotion of an all-encompassing preservation strategy on the quantity, quality, and ecology of cultivated land [19]. As the health of cultivated land is the foundation of national food security, only by diagnosing the health of cultivated land, evaluating the causes of unhealthy cultivated land, and implementing the appropriate treatment can the issue of unhealthy cultivated land be resolved at its source [20,21]. According to academics, the cultivated land system’s health condition is a complete representation of the cultivated land system, and research on the cultivated land system’s health status may provide fresh concepts for its management, preservation, and sustainable use. As a result, some researchers have used the Soil Management Assessment Framework (SMAF), the Soil Health Comprehensive Assessment—Cornell Framework (CASH), the Meta—Analytic Hierarchy Process index (Meta—AHP), the Soil Indicator Assessment (SINDI), the Soil Health Index (SHI), and other methods to assess the state of cultivated land system health and optimize farmland management practices [18,21,22,23,24,25]. In addition, some academics have conducted an empirical study on the health state of the cultivated land system by building an evaluation index system based on several scales, including towns, counties, cities, and provinces [26,27]. In general, the current research has achieved fruitful results. However, due to the complexity and comprehensiveness of the research topic, there are still the following problems: First, the idea and meaning of the health of the cultivated land system have not been fully elucidated by the current study, and some studies only include the microcosmic assessment of the cultivated land itself, maternal health, and soil health; second, in the process of selecting the health evaluation indicators and building the evaluation system of the cultivated land system, most of them focus on social and economic indicators, while the macroecological and environmental indicators are relatively few. At the same time, some studies do not consider the collinearity between the evaluation indicators; third, the subjectivity of determining the right of evaluation indicators is too strong, and the grading of evaluation results has not established a set of objective and unified standards.

In light of this, and in accordance with the strategic objective of agricultural sustainable development, this paper constructs the “PSR-VOR” cultivated land system health evaluation index system with the 5 km × 5 km grid as the evaluation unit, uses the principal component analysis and critical method to determine the comprehensive weight of the evaluation index, and employs the GIS spatial analysis and mathematical statistics methods to investigate the spatiotemporal pattern and evolution law of cultivated land system health.

## 2. Materials and Methods

### 2.1. Research Methods

#### 2.1.1. Framework for Health Assessment of Cultivated Land System

One concentrated example of the coupling effect between people and the land is the system of cultivated land, which consists of both the natural ecological system and the social and economic system [28]. The stability and sustained growth of the cultivated land system and an essential assurance for local food security depend on the health of the cultivated land system [27]. From the viewpoint of humans, the health of a cultivated land system refers to the resistance and adaptability of the cultivated land system in the process of coping with natural or human stress, as well as its ability to maintain the stability of system structure, self-recovery, and regulation when the system is damaged [29], which is the concentrated expression of the functions of cultivated land system in realizing food production, ecological regulation, and environmental restoration [30].

**Figure 1:**
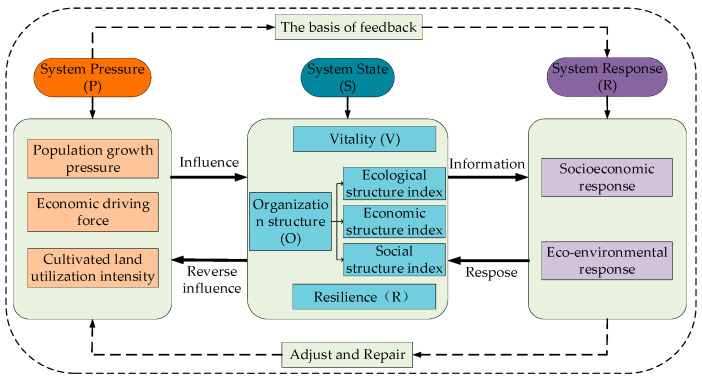
Framework of health assessment of a cultivated land system in the two lake plains based on PSR-VOR model.

To reasonably assess the health of a cultivated land system, identify the causal relationship between system elements, analyze the operation mechanism between different indicators, and explore the causal chain between natural ecological elements and socio-economic elements, this paper proposes to build a cultivated land system health diagnosis model (Figure 1) combining the PSR (Pressure-State-Response) model and the VOR (Vigor-Organization-Resilience) model on the basis of the research on the indicator system of cultivated land system health evaluation at home and abroad [31,32]. The system of cultivated land is a complicated one that is controlled by humans and is not an unadulterated part of the natural environment [27]. The evaluation of the health status of the cultivated land system needs to comprehensively screen the ecological, environmental, social, economic, and other attribute indicators related to the cultivated land from the meso and macro scales [25,30]. Under the principle of scientific, comprehensive, systematic, and feasible indicator selection, first, select indicators can completely and accurately reflect the health status of the cultivated land system; second, it is relatively easy to obtain data and the calculation amount of indicators is relatively reasonable; third, according to the status quo of cultivated land use and the changing characteristics of cultivated land landscape patterns in the two lake plains, a practical indicator system of cultivated land system health evaluation is constructed to reflect the pertinence and comprehensiveness of cultivated land system health evaluation.

(1) System pressure (P) is the term used to describe the load effect of the ecological environment of the cultivated land system induced by the stress of human social and economic activities and natural environmental factors [33,34], which is characterized by population growth pressure, economic driving force, and cultivated land utilization intensity.

First, this paper uses population density, urbanization rate, per capita gross domestic product, economic density, and other indicators to illustrate the impact of population increase, urbanization, and industrialization on the cultivated land system [35] by using ArcGIS software (v.10.7, ESRI, Redlands, CA, USA) to generate vector data from the statistical data of relevant indicators of 40 counties and cities in the two lake plains from 2000 to 2019, superimpose it on 2790 grids, count the total land area of counties and cities included in each grid in different years, and then use the area weighting method to calculate the index data of each grid in different periods.

(1) $$Y_{i}=\overset{n}{\underset{i=1}{\sum }}\overset{n}{\underset{j=1}{\sum }}a_{ij}X_{j}/\overset{n}{\underset{j=1}{\sum }}A_{j}$$

where: $Y_{i}$ is the index value of the *i*-th grid; $a_{ij}$ is the total land area of the *j*-th county and city included in the *i*-th grid; $X_{j}$ is the index data of the *j*-th county and city; $A_{j}$ is the total land area of the *j*-th county.

Second, this paper uses indicators such as land reclamation rate, pesticide application intensity, and agricultural fertilizer application intensity to describe the regional cultivated land use intensity and the pollution and damage to the cultivated land use environment [36,37] by using ArcGIS software (v.10.7, ESRI, Redlands, CA, USA) to generate vector data from the statistical data of relevant indicators of 40 counties and cities in the two lake plains from 2000 to 2019, superimpose it on 2790 grids, count the cultivated land area of counties and cities included in each grid in different years, and then use the area weighting method to calculate the index data of each grid in different time periods.

(2) $$Z_{i}=\overset{n}{\underset{i=1}{\sum }}\overset{n}{\underset{j=1}{\sum }}a_{ij}F_{j}/\overset{n}{\underset{j=1}{\sum }}A_{j}$$

where: $Z_{i}$ represents the index value of the *i*-th grid; $a_{ij}$ is the cultivated land area of the *j*-th county and city included in the *i*-th grid; $F_{j}$ is the index data of the *j*-th county and city; $A_{j}$ is the cultivated land area of the *j*-th county.

(2) A system state refers to the health state of the cultivated land system when it is affected by various natural or human stresses [38,39], which is mainly characterized by vitality, organizational structure (ecological structure index, economic structure index, and social structure index), and resilience.

First, the *NPP* and *NDVI* values of cultivated land can effectively reflect the important parameters of crop growth, nutrition information, and crop yield. In this study, the cultivated land productivity and normalized vegetation index are used to describe the comprehensive grain production capacity and the ability to ensure food security and stability [40,41]. Firstly, the average *NPP* and cultivated land area of 2790 grids from 2000 to 2019 are extracted by using the zoning statistics and superposition analysis tools in ArcGIS software (v.10.7, ESRI, Redlands, CA, USA), and then the total cultivated land productivity of each grid in different years is calculated.

(3) $$P_{i}={\bar{NPP}}_{i}\times A_{i}$$

where: $P_{i}$ is the total cultivated land productivity of the *i*-th grid, and its unit is t/(hm^2^·a); ${\bar{NPP}}_{i}$ is the average *NPP* value of the *i*-th grid, indicating the productivity of cultivated land per unit area, and its unit is gC/m^2^/yr; $A_{i}$ is the total area of cultivated land in the *i*-th grid. In addition, the average value of the Normalized Difference Vegetation index (*NDVI*) of the grid is extracted as the evaluation index data of different grids.

Second, since it is challenging to directly measure the ecological structure index, this study uses the biological abundance index in the technical specification for evaluation of ecological environment conditions (HJ/t192-2015) to measure the level of biodiversity in the study area [42,43], which is applied to demonstrate the stability and sustainability of the cultivated land system structure.

(4) $$I_{bio}=A_{bio}\overset{n}{\underset{i=1}{\sum }}\frac{0.11\times a_{i}}{A_{i}}\;\;$$

where: $A_{bio}$ represents the normalization coefficient of biological abundance, and the reference value is 511.2642131067; $a_{i}$ is the cultivated land area of the *i*-th grid; $A_{i}$ represents the total area of the *i*-th grid.

Third, the proportion of total agricultural output value and the proportion of agricultural employees are adopted in this study to reflect the supporting role of agricultural production activities and agricultural employees on the structural stability and sustainability of the cultivated land system [5,44]. Refer to Formula (1) for the calculation process of these two indexes.

Fourth, the resilience index can reflect the anti-interference ability and adaptability of the cultivated land system to maintain the stability of the system structure in the face of various stresses [45]. The per capita cultivated land area index is used to reflect the recovery degree of the cultivated land system. After strengthening the response mechanism and response ability and enhancing the system vitality and self-healing ability, the system will tend to be stable, the loss of high-quality cultivated land will be gradually reduced, and the cultivated land area will gradually increase [46]. Refer to Formula (2) for the calculation process of per capita cultivated land area index.

(3) System response (R) refers to the use of management, economy, policy, and other means to promote the rational use of cultivated land and promote sustainable and healthy development in order to cope with the current situation and the stress faced by the cultivated land system [47,48].

First, this paper uses the indicators of per capita grain occupancy, per capita net income of farmers, agricultural mechanization level, and agricultural power intensity to reflect the socio-economic response ability, adaptability, and coping ability of the cultivated land system when facing various stresses [49]. Refer to Formula (2) for the calculation process of these four indicators.

Second, since it is difficult to directly measure the environmental ecological response capacity, this study characterizes the environmental ecological response capacity with the help of the functions of climate regulation, waste treatment, soil conservation, and biodiversity maintenance in ecosystem services [50]. Using the equivalent factor method established by Xie Gaodi et al. [51], the ecological service value of the two lake plains is calculated. The ecological service value of other ecosystems is compared to the agricultural food production service value by assuming that the economic value of the national average 1 hm^2^ farmland food production equals 1. The following equation determines the entire ecological service value of the evaluation unit and the economic value of the agricultural food production function (Table 1) [52].

(5) $$E_{j}=1/7\overset{n}{\underset{i=1}{\sum }}\frac{a_{i}P_{i}Q_{i}}{A}\;$$

where: $E_{j}$ represents the economic value of the food production function provided by the farmland ecosystem per unit area (yuan/hm^2^); $a_{i}$ represents the area of the *i*-th crop; $P_{i}$ represents the average market price of the *i*-th crop (yuan/kg); *i* represents the *i*-th crop; $Q_{i}$ represents the unit yield of the *i*-th crop (kg/hm^2^); *A* represents the total area of grain crops.

According to the data of *Hunan Rural Statistical Yearbook*, *Hubei Rural Statistical Yearbook*, and *China Yearbook Agricultural Product Price Survey*, the average unit yield of major grain crops in the two lake plains from 2000 to 2019 was calculated to be 6546.377 kg/hm^2^ and the market average price was 2.76 yuan/kg. The economic value of grain output in the farmland ecosystem of the two lake plains was calculated to be about 2581.143 kg/hm^2^. The service value coefficient table of farmland ecosystem per unit area in the two lake plains (Table 1) is revised, and finally, the ecological service value of different grids in the two lake plains from 2000 to 2019 is calculated.

This study employs the principal component analysis technique to conduct statistical analysis on the evaluation indexes of the health state of the cultivated land system in the two lake plains, which is based on the PSR-VOR evaluation index framework. The KMO is 0.655 (greater than 0.6) and passes the Bartlett sphericity test (*p* < 0.05). The indicators are screened according to the variance interpretation rate and load coefficient. Finally, the cultivated land system health evaluation indexes are optimized from 29 to 19, 81.13% of the information is characterized by 65.51% of the indicators, the influence of subjective factors is effectively reduced, and the 19 evaluation indexes can fully reflect the health status of the cultivated land system (Table 2).

#### 2.1.2. Data Standardization and Indicator Weight Determination

According to their attributes, the health evaluation indicators can be categorized as positive indicators and negative indicators. The higher the value of the positive indicator, the better the health condition, while the higher the value of the negative indicator, the worse the health condition. It is required to normalize the original data of assessment indicators, owing to differences in size and magnitude across various indicators. The formula is as follows:

(6) $$Z_{ij}=\frac{x_{ij}-\min x_{ij}}{\max x_{ij}-\min x_{ij}}$$

(7) $$Z_{ij}=\frac{\max x_{ij}-x_{ij}}{\max x_{ij}-\min x_{ij}}$$

where: $Z_{ij}$ and $x_{ij}$ represent the standardized value and original value of the *j*-th single index in the *i*-th year; $\max x_{ij}$ and $\min x_{ij}$ respectively represent the maximum and minimum values of the *j*-th single indicator in all years.

Various assessment indices contribute differently to the health of the cultivated land system in the research region. This research uses the principal component analysis [53] and the critical weight technique [54,55] to establish the objective weight of the evaluation indicators and then calculate the average of the two objective weight results to get the final weight of the evaluation indicators.

#### 2.1.3. Evaluation Method of Cultivated Land System Health

The comprehensive index method is adopted in this study. Based on the standardized value and comprehensive weight of the selected evaluation indexes, the cultivated land system health index (*CLSHI*) of each evaluation unit within the study area is calculated by the weighted summation method. The formula is:

(8) $$CLSHI=\overset{n}{\underset{i=1}{\sum }}X_{i}\times w_{i}\;\;\;\;\;$$

where: *CLSHI* represents the cultivated land system health index; $X_{i}$ represents the attribute value after normalization of the *i*-th evaluation index; $w_{i}$ represents the combined weight of the *i*-th post screening evaluation index.

For the categorization of the health grade, there is currently no consistent standard. The cultivated land system health index of 2790 grids in the two lake plains from 2000 to 2019 is classified by using the natural discontinuity classification method (Jenks) in ArcGIS software (v.10.7, ESRI, Redlands, CA, USA), and the cultivated land system health index is divided into 5 categories, so as to reflect the health status in the two lake plains (Table 3).

#### 2.1.4. Spatial Autocorrelation Analysis

In order to investigate the geographical correlation of a variable and the strength of that correlation, spatial autocorrelation analysis is separated into two categories: global spatial autocorrelation and local spatial autocorrelation [56]. The global spatial autocorrelation is primarily used to examine the degree of aggregation of a certain attribute value in the global space and can be used to define the spatial autocorrelation of the regional cultivated land system health index as a whole. The formula is:

(9) $$I=n\overset{n}{\underset{i=1}{\sum }}\overset{n}{\underset{j=1}{\sum }}W_{ij}\left(Z_{i}-\bar{Z}\right)\left(Z_{j}-\bar{Z}\right)/\overset{n}{\underset{i=1}{\sum }}\overset{n}{\underset{j=1}{\sum }}W_{ij}\overset{n}{\underset{i=1}{\sum }}{\left(Z_{i}-\bar{Z}\right)}^{2}$$

where: $Z_{i}$ and $Z_{j}$ denote the evaluation values of the *i*-th and *j*-th evaluation units, respectively; $W_{ij}$ represents a binary distance or adjacency space weight matrix; *n* represents the total number of evaluation units.

#### 2.1.5. Cold and Hot Spot Analysis

Cold and hot spot analysis is mainly used to identify the spatial concentration of the estimated values in cold and hot spots in different local spaces. It is an analysis method of local autocorrelation characteristics [57]. The function formula of Getis-Ord $G_{i}^{*}$ is as follows:

(10) $$G_{i}^{*}=\overset{n}{\underset{i=1}{\sum }}w_{ij}\left(d\right)x_{i}/\overset{n}{\underset{j=1}{\sum }}x_{j}$$

(11) $$Z\left(G_{i}^{*}\right)=\frac{G_{i}^{*}-E\left(G_{i}^{*}\right)}{\sqrt{Var\left(G_{i}^{*}\right)}}$$

where: $G_{i}^{*}$ is the statistic of the *i*-th grid, and the correlation degree between the *i*-th grid and the *j*-th grid is obtained through distance weight calculation; $w_{ij}\left(d\right)$ represents a spatial adjacent weight matrix within the range of distance *d*; $E\left(G_{i}^{*}\right)$ and $Var\left(G_{i}^{*}\right)$ were mathematical expectation and variance of $G_{i}^{*}$. Among them, if $Z\left(G_{i}^{*}\right)$ is significantly positive, it indicates that the values around the *i*-th grid are relatively high and belong to the hot spot area.

### 2.2. Research Area

**Figure 2:**
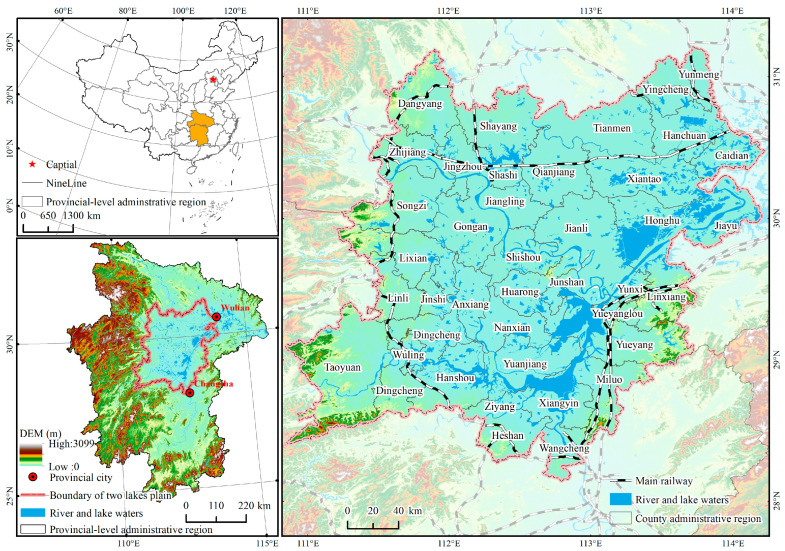
Topographic map of the two lake plains and distribution map of County Administrative Region.

The two lake plains defined in this study span 110°51′–114°22′ E and 27°58′–31°12′ N, mainly including 40 counties and urban areas, with a total area of about 63,700 km^2^ (see Figure 2). The two lake plains are an important grain production functional region and agricultural product protection area in China, and they are one of the places with a high degree of agricultural economic growth. According to the primary findings of the National Cultivated Land Quality Grade Update and Assessment in 2016, which was released by the Chinese Ministry of Natural Resources, the provinces of Hubei, Hunan, and Guangdong included the majority of China’s good land. With a combined size of 35,201 million hectares, these three provinces made up 90.28 percent of all the country’s excellent land, with Hubei housing the bulk of the nation’s remarkable land. Roughly 17.3242 million tons of grain were produced in the two lake plains region in 2019, making up about 30.39 percent of the total grain production in the provinces of Hunan and Hubei.

### 2.3. Data Resource

The indicator data of CLSH consist primarily of natural environment and socio-economic data (including vector and raster data) pertaining to the cultivated land system: (1) Land use/cover data from 2000 to 2015. This dataset is part of the Chinese Academy of Sciences’ 1980 data (accessed on 19 January 2021, https://www.resdc.cn) collection on land use and cover change in China. Landsat TM/ETM pictures and HJ-1 and CBERS-2 satellite data are utilized as the primary data sources. Since the 1980s, 1 million pieces of land use data have been extracted every five years using a quick extraction technique based on remote-sensing information and human-computer interaction. The overall precision of the first and second classes of land use is 94.30% and 91.20%, respectively [58]. (2) Land use/cover data in 2019. Landsat-8 OLI remote sensing pictures are the primary source of data. There are eight landscape photos (strip numbers 123–125, lines 38–40) with a 30 m spatial resolution and less than 5% cloud coverage. The photographs have been obtained from the website for geospatial data clouds (accessed on 1 February 2021, https://www.gscloud.cn). ENVI 5.3 is utilized for radiometric calibration, atmospheric correction, geometric accuracy correction, image mosaicking, and image cropping. According to the CNLUCC data classification system [59], the classification process is supervised by the support vector machine (SVM) method. The first level is separated into six groups based on the land resources and their utilization characteristics: cultivated land, forest land, grassland, water area, building land, and unused land. According to the natural features of land resources, the secondary classification is separated into 25 categories, and the accuracy of the classification results has been tested using field verification points. The classification accuracy of each kind and the total classification accuracy of the results of image supervision are greater than 87%, and the kappa coefficient is greater than 0.9. The categorization accuracy meets the research standards. (3) The data on digital elevation, lakes and rivers, town points, and administrative boundaries of the two lake plains are from the National Basic Geographic Information Center of China (accessed on 16 December 2020, https://www.webmap.cn/main.do?method=index). (4) Panel statistics. It mainly includes the *Statistical Yearbook of Hunan Province,* the *Statistical Yearbook of Hubei Province,* the *Rural Statistical Yearbook of Hunan Province,* the *Rural Statistical Yearbook of Hubei Province,* the *Yearbook of China Agricultural Product Price Survey,* and the statistical yearbook of prefecture-level cities in the study area from 2000 to 2019. Sources and spatial resolution of these data are shown in Table 3.

In order to ensure the accuracy of the evaluation index data, first, the vector and grid data are projected and transformed, and all are unified to CGCS2000_3_Degree_GK_Zone_38 projection coordinate system and GCS_China_Geodetic_Coordinate_System_2000 geographical coordinate system to check and correct topological errors of vector data. Second, in the process of superposition analysis and assignment of vector data, when the area of the planar statistical unit is larger than the evaluation unit, the statistical unit is divided with the evaluation unit as the minimum unit, and the comprehensive value of the evaluation unit is calculated by the area weighting method. Third, in the process of statistical analysis and assignment of grid data, the average value of 2790 grid (5 km × 5 km) data is calculated by using the partition statistical tool of ArcGIS software (v.10.7, ESRI, Redlands, CA, USA).

## 3. Results

### 3.1. Spatiotemporal Variation Characteristics of Different Cultivated Land System Indices

#### 3.1.1. Analysis of Time Series Change of Cultivated Land System Index

**Figure 3:**
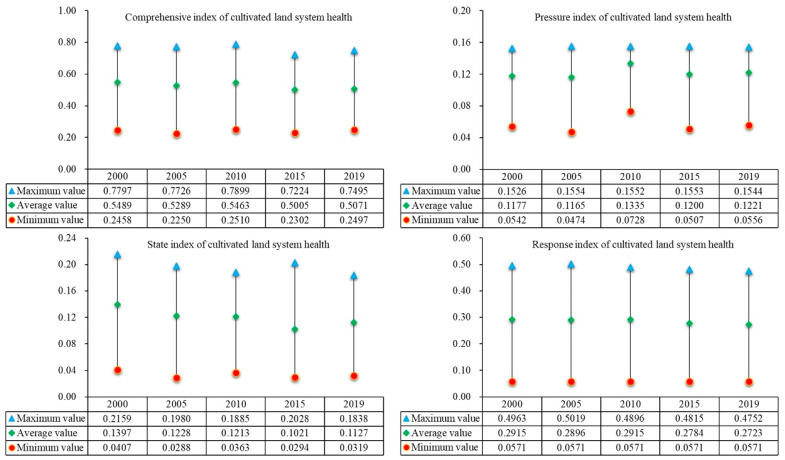
Change characteristics of cultivated land system index in the two lake plains from 2000 to 2019.

According to the relevant formulas and evaluation index data, the pressure, state, and response index values of 2790 grids in the two lake plains from 2000 to 2019 are calculated and the maximum, minimum, and average values are obtained. The cultivated land system health index has fluctuated and declined over the past two decades, as illustrated in Figure 3. The maximum and average values of the cultivated land system health index go down by 0.0302 and 0.0418, respectively, from 0.7797 and 0.5489 in 2000 to 0.7495 and 0.5071 in 2019. The minimum value of health index increases from 0.2458 in 2000 to 0.2497 in 2019, an increase of 0.0039. In general, the health status shows a fluctuating downward trend from 2000 to 2019. The stronger the beneficial influence of human activities on the health of the cultivated land use system, the lower the pressure index. From 2000 to 2019, the average pressure index increases by 0.0044, and the pressure level shows a fluctuating upward trend as a whole. The stronger the status index, the greater the influence of human activities on the health of the cultivated land system. From 2000 to 2019, the average state index decreases by 0.027, and the state level shows a fluctuating downward trend. The stronger the response index, the greater the beneficial influence of human activities on the health of the cultivated land system. From 2000 to 2019, the average response index decreases by 0.0192, and the overall response level shows a fluctuating downward trend.

#### 3.1.2. Analysis on Spatial Change of Cultivated Land System Index

The hot spot analysis tool in ArcGIS software (v.10.7, ESRI, Redlands, CA, USA) is used to calculate the Getis-Ord $G_{i}^{*}$ index of 2790 grids in the two lake plains from 2000 to 2019, and the natural discontinuity classification method (Jenks) is used to classify the $G_{i}^{*}$ value into 5 categories from high to low and further analyze the evolution trend of the cold and hot spot spatial patterns of the pressure, state, and response index.

**Figure 4:**
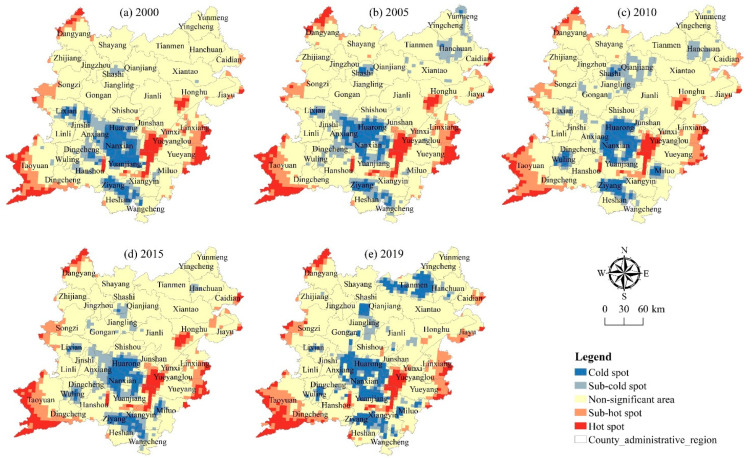
Spatial pattern of pressure index and cold hot spot of cultivated land system in the two lake plains from 2000 to 2019.

Figure 4 shows that the distribution of hot spots and sub-hot spots of the pressure index has little change and is relatively scattered, showing a general distribution trend of “small agglomeration and large dispersion”, mainly distributed in the plain areas greatly affected by rivers and lakes and the areas with relatively general cultivated land resource endowment conditions at high altitude. Some of them are mainly distributed along the lakes and rivers in Honghu, Junshan, Yueyanglou, and other areas, and some are concentrated in Dangyang, Songzi, Taoyuan, Linxiang, and other areas with relatively high altitude around the two lake plains. From 2000 to 2019, the distribution of cold and hot spots in the pressure index in the two lake plains is relatively concentrated, exhibiting a spatial pattern in which the low-value cold spots center on “Huarong-Anxiang-Nanxian-Yuanjiang” are gradually distributed around, mainly concentrated in Lixian County, Tianjin city, Huarong, Nanxian County, Yuanjiang and Ziyang.

**Figure 5:**
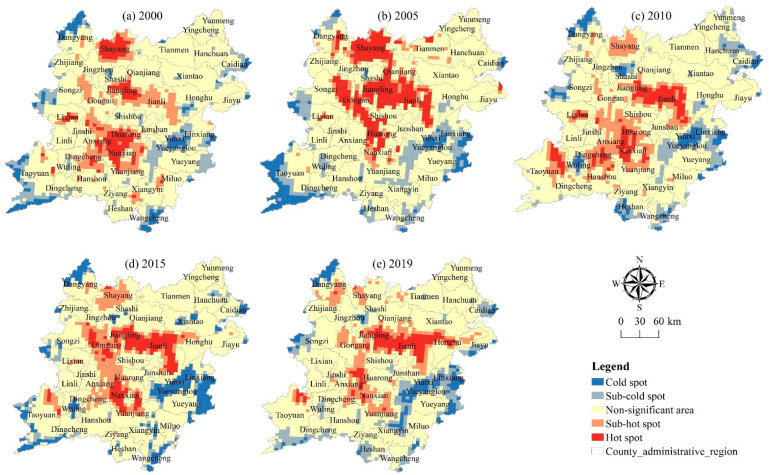
Spatial pattern of cold and hot spots of status index in the two lake plains from 2000 to 2019.

As shown in Figure 5, the hot spots and sub-hot spots of status index show a trend of shifting from the southwest to the northwest, forming an “A” shaped hot spot cluster distribution area with “Jianli-Jiangling-Gongan-Huarong-Nanxian-Yuanjiang” as the core. The distribution of cold spots and sub-cold spots of the pressure index is relatively scattered, showing a trend of shifting to the southwest and southeast, forming the spatial distribution characteristics of “small agglomeration and large dispersion” cold spots centered on “Yunxi-Yueyanglou-Linxiang”.

**Figure 6:**
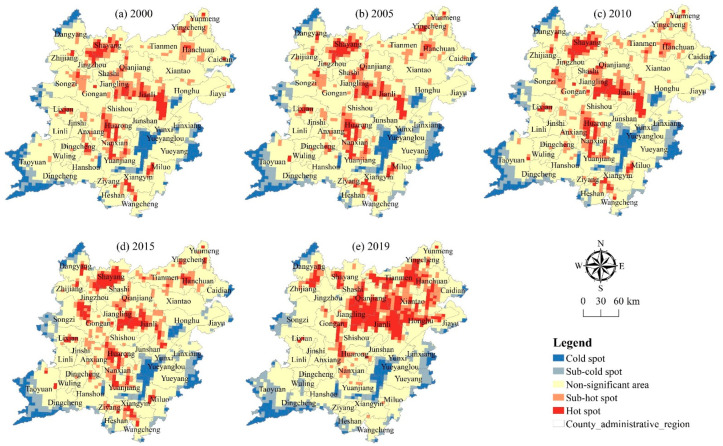
Spatial pattern of cold and hot spots of response index in the two lake plains from 2000 to 2019.

As shown in Figure 6, the hot spots and sub-hot spots of the response index show a multi-center strip-type distribution trend, forming a strip-type hot spot distribution feature with Shayang, Jianli Jiangling, and Huarong Nanxian as the core. The distribution of cold spots and sub-cold spots of the pressure index is not obvious, and it generally presents the distribution characteristics of “small agglomeration and large dispersion”, which is concentrated in the southeast and southwest of the two lake plains, forming a cluster-like spatial distribution pattern of “small agglomeration” in the cold spot areas of Yueyanglou, Linxiang, Taoyuan, Songzi, and Honghu.

### 3.2. Temporal Difference Characteristics of Health Status of Cultivated Land System

In order to further analyze the differences in health status at different stages, the field calculator tool in ArcGIS software (v.10.7, ESRI, Redlands, CA, USA) is used to classify the health index of 2790 grids in the two lake plains from 2000 to 2019. The difference of the health level in different periods is obtained. Wherein, if the level difference is positive, it indicates that the health status tends to improve, and the greater the value, the more obvious the improvement; if the level difference is negative, it indicates that the health status tends to deteriorate, and the greater the value, the more obvious the deterioration. On this basis, Kriging interpolation method is used for spatial interpolation and divided into five categories to analyze the change direction and regional difference of health status in different stages from 2000 to 2019.

**Figure 7:**
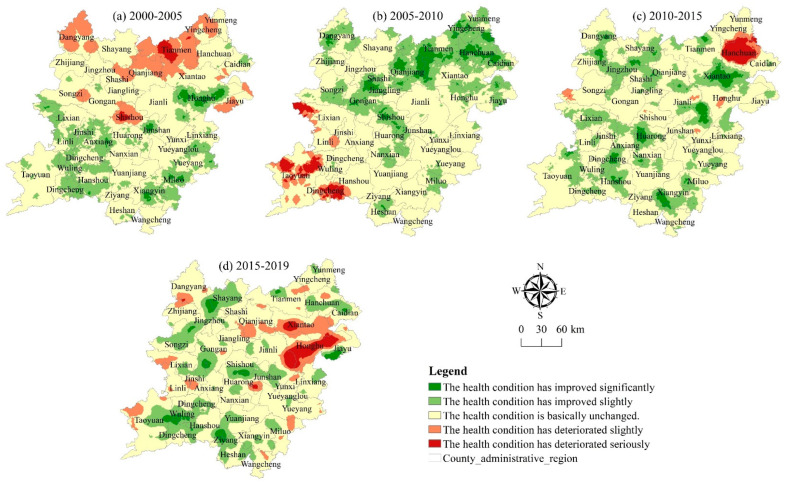
Change direction of cultivated land system health in the two lake plains from 2000 to 2019.

Figure 7 and Table 4 demonstrate a declining trend in the extent of Class I and Class II land in the two lake plains from 2000 to 2005, with a decrease of 168,500 hm^2^ and 15,900 hm^2^; respectively, with a decrease of 9.56% and 1.28%. The area of cultivated land with Class III, Class IV, and Class V health status has increased. Among them, the area of cultivated land with Class III has increased the most, reaching 58,400 hm^2^, while the area of cultivated land with Class IV has increased the most, accounting for 80.33%. During this period, the areas with obvious or slight deterioration in the health of the cultivated land system are mainly distributed in the middle and north of the two lake plains. The closer it is to the cities and towns, the higher the input and utilization. The interference of human activities on the regional cultivated land system will gradually increase, resulting in the reduction of the health level.

The area of Class I cultivated land shows a negative trend between 2005 and 2010, declining by 593,900 hm^2^. The area of cultivated land with Class II, Class III, Class IV, and Class V shows an upward trend. Among them, the area of cultivated land with Class I increases the most, reaching 384,300 hm^2^, an increase of 31.13%. During this period, the areas where the health status of the cultivated land system has been significantly improved or slightly improved are mainly distributed in the northeast of the two lake plains. These areas have gradually changed the mode of cultivated land utilization, adjusted the agricultural industrial structure, and improved the level of intensive utilization of cultivated land. When decision-makers and operators respond positively to the health status and promote the development of characteristic planting and breeding industries, the traditional agricultural economic development model’s negative impact on the health of the cultivated land system is effectively mitigated.

From 2010 to 2015, the area of cultivated land with Class I shows a downward trend, with a decrease of 647,400 hm^2^, a decrease of 64.68%. The area of cultivated land with Class II, Class III, Class IV, and Class V shows an upward trend. Among them, the area of cultivated land with Class III increases the most, reaching 180,300 hm^2^, while the area of cultivated land with Class IV increases the most, reaching 53.92%. During this period, the areas where the health status of the cultivated land system deteriorated significantly or slightly are mainly located in Hanchuan, Yunmeng, and other areas in the northeast of the two lake plains. These areas are relatively close to Wuhan city. The urbanization drive has a certain impact on the cultivated land use mode and agricultural development mode in this area, especially in the process of developing fruit, vegetables, and other facilities agriculture to meet the demand of agricultural products in large cities, as the unreasonable large-scale use of pesticides and fertilizers will increase the external pressure.

From 2015 to 2019, the area of cultivated land with Class I and Class II in the two lake plains shows a downward trend, with a decrease of 171,200 hm^2^ and 227,200 hm^2^, respectively, with a decrease of 48.45% and 12.54%. However, the area of cultivated land with Class III, Class IV, and Class V has increased. During this period, the areas where the health of the cultivated land system have deteriorated significantly or slightly are mainly located in Xiantao, Honghu, and other areas in the northeast of the two lake plains. As urbanization and industrialization have advanced, a high demand for land has resulted from the concentration of people, businesses, and other factors. The unreasonably intensive use of cultivated land and agricultural pollution have negatively impacted the system’s ability to support cultivation; the cultivated land in some areas is easily affected by floods, soil erosion is serious, ecological environment sensitivity is strong, and the ability of the cultivated land system to resist external interference is poor.

In general, from 2000 to 2019, the area of cultivated land with Class I in the two lake plains decreases year by year, with a net decrease of 1.5812 million hm^2^, a decrease of 89.67%. The area of cultivated land with Class I, Class III, Class IV, and Class V has increased. The net increase area of cultivated land with Class III is at most 428,200 hm^2^, while the increase area of cultivated land with Class III is 264.96%. On the whole, the health level of the cultivated land system in the two lake plains tends to be stable after decreasing year by year. In the future, it will be required to pay special attention to the deteriorating trend of the local cultivated land system’s health state and to increase the monitoring and early warnings of the regional cultivated land system’s health condition.

### 3.3. Spatial Difference Characteristics of Health Status of Cultivated Land System

#### 3.3.1. Spatial Distribution of Health Status of Cultivated Land System

**Figure 8:**
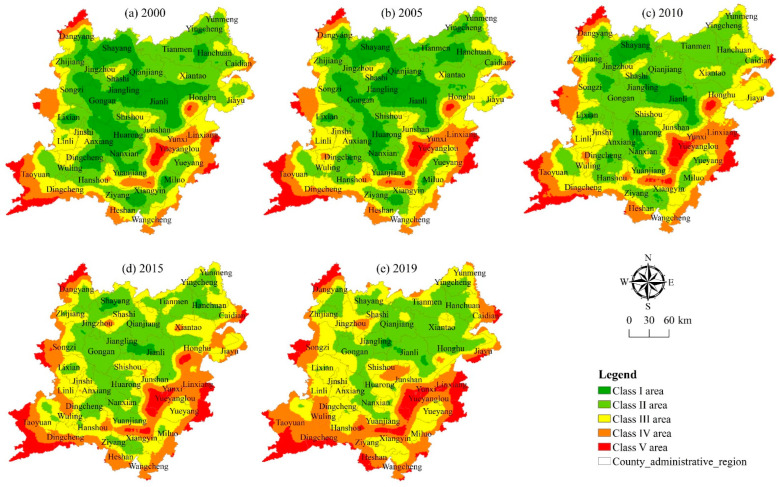
Spatial distribution of cultivated land system health in the two lake plains from 2000 to 2019.

Using the health index values of 2790 grids in the two lake plains from 2000 to 2019 for spatial interpolation, the Kriging interpolation technique in the ArcGIS software (v.10.7, ESRI, Redlands, CA, USA) is split into five groups based on the classification standard. It can be seen from Figure 8 that from 2000 to 2019, the spatial pattern of the health status in the two lake plains changes greatly, and the spatial heterogeneity is relatively significant. The health level generally shows a distribution characteristic of gradually decreasing from the northeast to the southwest and southeast.

In the past 20 years, the Class I areas in the two lake plains are mainly distributed in the southern area of the two lake plains with “Jinshi-Huarong-Anxiang-Nanxian-Yuanjiang” as the core and the northern area of the two lake plains with “Gongan-Jiangling-Jianli-Qianjiang” as the core. These areas have good water and heat matching, fertile soil, good agricultural foundation, high agricultural development level, and very complete structure and function. In the five periods from 2000 to 2019, the cultivated land area of Class I area is 1,763,400 hm^2^, 1,594,900 hm^2^, 100,900 hm^2^, 353,500 hm^2^, and 182,200 hm^2^ respectively, accounting for 46.83%, 43.04%, 27.19%, 9.88%, and 5.6% of the total cultivated land area. In general, Class I area shows a decreasing trend year by year.

The Class II area in the two lake plains is mainly distributed in layers around the Class I area, showing a trend of contraction in the southwest and expansion in the northeast. The status and functions in these areas are basically complete, there is no system abnormality, the system vitality and self-healing ability are strong, and the cultivated land system is in a relatively stable and sustainable state. In the five periods from 2000 to 2019, the Class II area is 1,250,600 hm^2^, 1,234,600 hm^2^, 1,618,900 hm^2^, 1,811,900 hm^2^, and 1,584,600 hm^2^ respectively, accounting for 33.21%, 33.32%, 43.97%, 50.65%, and 48.67% of the total cultivated land area (Table 5) and showing an increasing trend year by year.

Class III areas in the two lake plains are concentrated in the surrounding areas of cities and towns, showing a trend of gathering from the northwest, southwest, and southeast of the two lake plains to the middle. These areas are relatively affected by the expansion of urbanization. They are suburban facilities agricultural production bases. The economic benefits of cultivated land utilization are relatively high. The structure and function of the regional cultivated land system are still complete, but the system vitality and self-healing ability are general. If a few system abnormalities have occurred, the system can still maintain a stable and sustainable state. In the five periods from 2000 to 2019, the Class III area is 522,000 hm^2^, 580,500 hm^2^, 73.27 hm^2^, 913,100 hm^2^, and 950,300 hm^2^ respectively, accounting for 13.86%, 15.67%, 19.9%, 25.53%, and 29.19% of the total cultivated land area (Table 5) and showing an increasing trend year by year.

Class IV areas in the two lake plains are mainly distributed in the surrounding areas of cities and towns, river lake plain areas, or mountainous and hilly areas, showing a trend of gathering to the southwest and southeast. The factors affecting the health of the cultivated land system in these regions are complex, being affected by urbanization expansion, natural disasters, water and soil loss, etc. The structure and function of the regional cultivated land system are not very complete, and the system vitality and self-healing ability are poor. A large number of system abnormalities have occurred, and the system has begun to deteriorate. From 2000 to 2019, the Class IV area in the five periods is 201,300 hm^2^, 244,000 hm^2^, 26.87 hm^2^, 405,100 hm^2^, and 434,900 hm^2^, respectively, accounting for 5.35%, 6.59%, 7.3%, 11.33%, and 13.36% of the total cultivated land area (Table 5) and showing an increasing trend year by year.

Class V areas in the two lake plains are mainly distributed along the river and the lake or concentrated in the mountainous and hilly areas around the edge of the two lake plains, forming a concentrated distribution area in the southeast, northwest, and southwest of the two lake plains. The health of the cultivated land system in these areas is affected by human activities and natural environment such as reclaiming land from lakes, returning farmland to lakes, returning farmland to forests, soil erosion, etc. The health structure of the regional cultivated land system is damaged, the system vitality and self-healing ability are poor, and the system function is degraded to a certain extent. From 2000 to 2019, the Class V area is 28,400 hm^2^, 51,300 hm^2^, 6606 hm^2^, 93,400 hm^2^, and 103,900 hm^2^ respectively, accounting for 0.76%, 1.39%, 1.65%, 2.61% and 3.19% of the total cultivated land area (Table 4) and showing an increasing trend year by year.

In general, from 2000 to 2019, the classification areas are mainly Class I and Class II. The cultivated land area of the two levels accounts for more than 54.27% of the whole region, indicating that the overall health status in the two lake plains has been at a good level during the study period, but the area of cultivated land with Class III and Class IV has increased rapidly. Future emphasis must be placed on monitoring the changes in the health state in these regions.

#### 3.3.2. Spatial Autocorrelation Analysis

**Figure 9:**
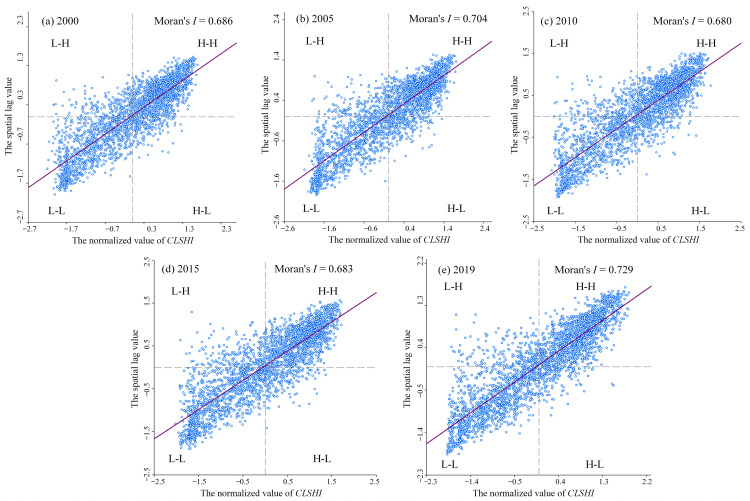
Moran’s *I* scatter diagram of *CLSHI* in the two lake plains from 2000 to 2019.

To assess the spatial convergence and agglomeration of the health status in the two lake plains, a global spatial autocorrelation analysis has been performed on the health index data of 2790 grids in the two lake plains from 2000 to 2019 using OpenGeoDa software, and a Moran’s *I* scatter diagram has been generated (Figure 9). From 2000 to 2019, the global Moran’s *I* values of the cultivated land system health index in the two lake plains are 0.686, 0.704, 0.68, 0.683, and 0.729, respectively. The global Moran’s *I* values in the five periods are all greater than 0, and through the significance test of the 5% confidence level, the overall shows a fluctuating upward trend, indicating that there is a significant spatial positive autocorrelation in the health index in each grid of the two lake plains, and that its spatial distribution was not random, there is an obvious trend of agglomeration and distribution of adjacent grids in space. In addition, from 2000 to 2019, the spatial concentration degree of the health index in the two lake plains exhibits a fluctuating trend, indicating that the change of the health index in the two lake plains is accelerated and the spatial heterogeneity of the health index is progressively increased.

**Figure 10:**
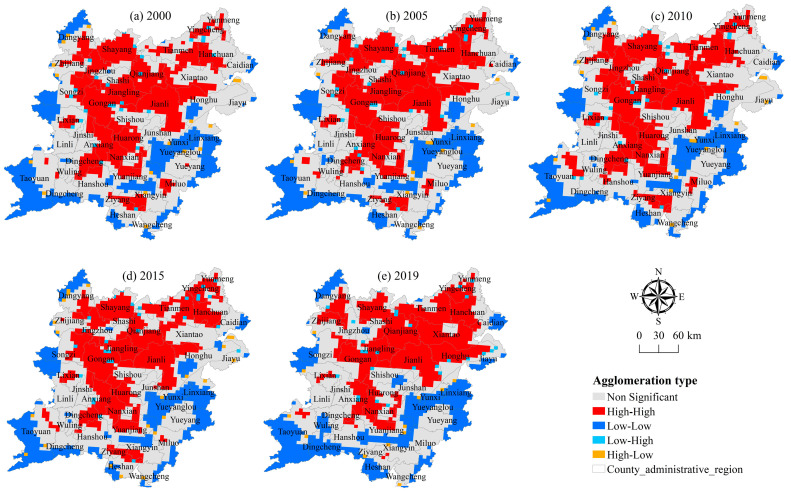
Local spatial autocorrelation diagram of *CLSHI* in the two lake plains from 2000 to 2019.

This study uses Opengeoda and ArcGIS software (v.10.7, ESRI, Redlands, CA, USA) to analyze and obtain the local spatial autocorrelation LISA map of the cultivated land system health index in the two lake plains from 2000 to 2019 in order to further analyze the spatial autocorrelation degree and spatial agglomeration mode of the cultivated land system health index between different grids. It can be seen from Figure 10 that from 2000 to 2015, the local spatial autocorrelation cluster area of the health index does not change significantly, showing a general trend of “high in the middle and low in the periphery”. Among them, the H-H cluster area of the health index forms a double strip distribution area. These areas have good water and soil conditions, high-standard farmland construction and land treatment, effectively ensuring the stability of grain production, stable structure and complete functions, and the health level is high. The L-L cluster area is distributed along the lake, river, and mountain area in a “cluster” shape. The cultivated land resources in these areas are relatively small, the input of cultivated land is insufficient, the ecological environment is sensitive, and it is vulnerable to the double effects of human activities and natural disasters. The cultivated land system is not strong in resisting risks and interference. From 2015 to 2019, the local spatial autocorrelation cluster area of the cultivated land system health index has changed significantly, showing a trend of high-value areas to the northeast and low-value areas to the southeast. Among them, the range of H-H cluster area is gradually reduced, showing a “cluster” cluster distribution, while the range of L-L cluster area is gradually expanded, showing a “strip” distribution along rivers, lakes, and mountains, and obviously expanding to the southwest and southeast of the two lake plains. In general, the distribution of high-value cluster areas and low-value cluster areas of the cultivated land system health index in the two lake plains from 2000 to 2019 do not change significantly, maintaining a basically stable spatial distribution pattern and showing a distribution characteristic of high in the north, low in the south, and decreasing from the middle to the outside.

### 3.4. Zoning Management of Health Status of Cultivated Land System

**Figure 11:**
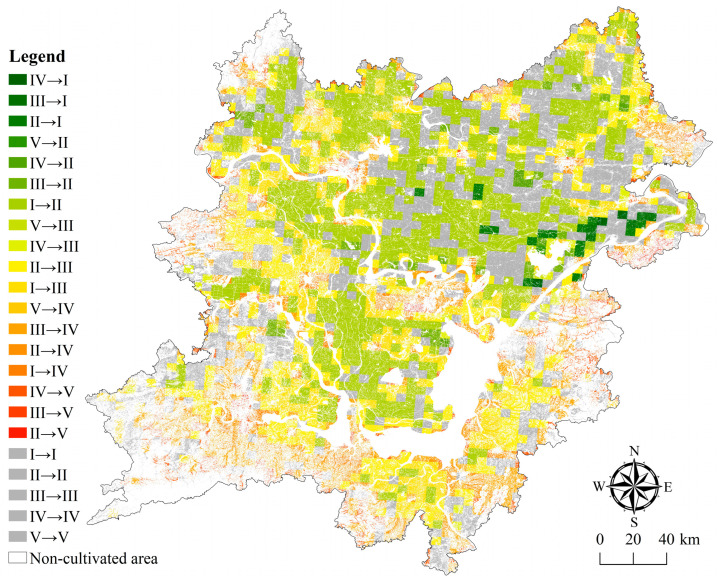
Spatial transformation diagram of cultivated land system health in the two lake plains from 2000 to 2019.

The purpose of this paper is to fully understand the coupling mechanism and action mechanism among the various elements of the cultivated land system, to classify and partition the health status of the cultivated land system in combination with the natural environmental conditions and social and economic development needs of the study area, and identify the key areas where the cultivated land protection and health status need to be improved, so as to provide reference for the formulation of plans and measures to improve the health status. As depicted in Figure 11, a comparison of the spatial difference and spatial transformation degree of the health status between 2000 and 2019 reveals that the improvement degree of the health status in the north of the two lake plains is obviously greater than that in the south, and that there are significant differences in the change direction of the cultivated land system’s health status in different regions. It can be seen from the results of the spatial transformation matrix in Table 6 that box 11 in this table indicates that this category actually exists in the transformation process. 11 indicates that in 2000 and 2019, it is considered a Class I and no conversion occurred. 52 indicates that the Class V in 2000 has been transformed into the Class II in 2019.

**Figure 12:**
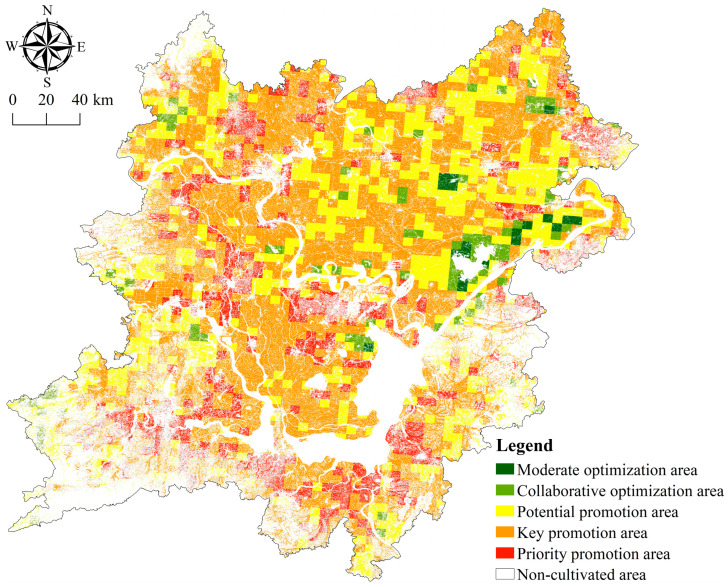
Spatial zoning of cultivated land system health in the two lake plains.

The spatial division is a process of the overall consistency of cultivated land system health status in a certain region and the differentiation of cultivated land system health status in different regions. In this paper, according to the change degree of the spatial transformation of the health status and the difference of the coupling degree, the 23 transformation types are divided into five health status zones (Figure 12 and Table 7); namely, the modern optimization area, the collaborative optimization area, the potential promotion area, the key promotion area, and the priority promotion area. There are differences in population growth, economic development, environmental ecology, etc. within each sub-district. The urgency of adjusting and controlling the health status from the moderately optimized area to the preferentially upgraded area has gradually increased. (1) The area of cultivated land in the moderately optimized area is 36,925.57 hm^2^, accounting for 1.12% of the total area of cultivated land in the whole area, which is mainly distributed in the northeast of the two lake plains. The health status in this area has improved significantly, with an overall improvement of two grades. (2) The area of cultivated land in the collaborative optimization area is 134,011.15 hm^2^, accounting for 4.07%. It is mostly dot and block shapes dispersed across the northeast, center, and southwest of the two lake plains. The health status in this sub-district has been slightly improved, and the overall level has been upgraded. (3) The cultivated land area in the potential promotion area is 911,500.41 hm^2^, accounting for 27.68%, mainly distributed in the northeast and southwest of the two lake plains in strips and blocks. The health status in this sub-district is relatively stable, with little overall change. However, there is still room for improvement in some areas where the health status is relatively general or poor. (4) The cultivated land area of the key promotion area is 1,781,765.18 hm^2^, accounting for 54.11%, which is mainly distributed in the northwest and southwest of the two lake plains. The health status in this sub district has slightly deteriorated, and the overall level has dropped by one level. (5) The area of cultivated land in the priority promotion area is 428,720.23 hm^2^, accounting for 13.02%, which is mainly distributed in strips along the rivers and lakes in the southeast and southwest of the two lake plains and the surrounding areas of the city. The health status in this sub district has deteriorated, obviously, and has dropped by two grades as a whole.

## 4. Discussion

### 4.1. Deficiency and Prospect

In the 21st century, the issues of food security and land pollution are major issues that have been widely concerned by the international community after the issues of population, resources, and ecology [60,61]. China’s two lake plains are a major grain-producing area and agricultural production base. As one of the most typical areas where human beings use cultivated land intensively to drive ecological environment changes, its cultivated land system evolution process has certain complexities and forerunners [62]. In the past two decades, as population and urbanization have increased, the problems of decreasing arable land, soil degradation, and agricultural pollution have gotten more severe [63,64]. Particularly, the excessive application and intensive use of chemical fertilizers have had a substantial effect on the health of the cultivated land system. The resulting risks of shallow tillage strata, apparent reduction of organic matter in surface soil, and excessive heavy metals in soil not only exacerbate the deterioration of the ecological environment of cultivated land, but also reduce grain yield, resulting in a decline in the quality and biodiversity of agricultural products and even endangering human health [27]. At present, the study on the ecological environment of cultivated land resources in the two lake plains mainly focuses on the agricultural ecological security [65], soil environmental quality [66,67], ecological security of farmland resources [68,69], ecosystem supply balance [50,70], and so on. However, there are relatively few evaluation studies on the health status of the cultivated land system in the two lake plains. Consequently, how to accurately evaluate and assess the physical, chemical, and biological aspects of soil has long been a central concern in the study of cultivated land system health. Currently, there is no one assessment method that can be used to evaluate all aspects of cultivated land system health, and it is difficult to quantify the synergy and interaction between these components [9].

Cultivated land system health assessment is an explanatory framework that evaluates the health condition of cultivated land systems by scoring the biological, chemical, and physical aspects of cultivated land in order to guide cultivated land utilization and crop production management [71]. The significance of the cultivated land health evaluation index at different scales is dynamic and must vary as index properties and resource management objectives for cultivated land change. Currently, the existing framework for assessing the health of cultivated land systems includes the spatial quantification of traditional indicators such as soil organic matter, pH value, soil bulk density, soil available phosphorus, and soil available potassium, with chemical indicators comprising the majority of all indicators [9]. Heavy metals are the most significant markers of agricultural soil among these [72]. Soil heavy metal contamination would not only result in soil deterioration and biodiversity loss, but would also damage human health via the food chain, whereas soil biodiversity is essential for boosting ecosystem processes and functions [73,74]. Simultaneously, an increasing number of scholars characterize biodiversity from the vantage point of cropland offering diverse ecological services [75]. With the advancement of remote-sensing technology, the application of near-infrared and mid-infrared technologies to monitor biodiversity will make the current farming system health assessment more practical and economical [9]. In addition, the construction of health indicators for the cultivated land system connected to climate change, such as greenhouse gas emissions and carbon sequestration, has been largely disregarded. Due to the fact that greenhouse gas emissions are dependent on changeable conditions (such as humidity and temperature), it is impossible to quantify the greenhouse gas flux in a given field or region using a single measurement of cropland [9]. Moreover, clarifying the context and goals of the health management of cultivated land systems is more valuable and significant. For instance, using nitrogen fertilizer to improve crop productivity can increase the emission of nitrous oxide, a potent greenhouse gas [76]. Consequently, there are significant disparities in the difficulty and complexity of selecting health indicators of cultivated land systems based on climate change or food production management for various aims.

In this paper, the improved “PSR-VOR” evaluation index system is applied to diagnose the health status of the cultivated land system in the two lake plains, and it is implemented spatially. It is helpful to identify the key areas where the cultivated land protection and health status need to be improved. To a certain extent, it can meet the requirements of protecting cultivated land according to local conditions and provide a reference for the study area to formulate plans and measures to improve the health status in different regions. However, there are still the following shortcomings: first, according to the construction method of the evaluation index system that adapts measures to local conditions and varies with time, the evaluation index system in different periods and regions will have different emphasis on the selection of indicators [69]. Cultivated land systems in different regions have different utilization modes, and the health problems encountered in the development process and the methods and means adopted to solve the problems are different [44]. Therefore, various areas must be given different weights when creating the assessment index system in order for it to accurately represent the current state of the health of the regional cultivated land system. In addition, for the same region, different periods indicate different development stages, and different development stages have different regional development objectives, cultivated land use modes and cultivated land use modes, so there are different emphases in the process of building the evaluation index system [77]. The second is the quantification of evaluation index data and the recurrence of scale. In this paper, the evaluation unit is set as the same size cell grid, which can effectively represent the spatial difference of cultivated land system health. This paper assumes that socio-economic data are related to land use types and other factors, which are not evenly distributed. For example, the population density on construction land in county-level administrative regions is generally greater than that of other land types. Although the evaluation units at the edge of the study area are incomplete, resulting in some deviation in the calculation results of these evaluation units, the cell grid area at the edge of such areas accounts for less than 3% of the total area and has little impact on the overall spatial pattern. On this basis, how to more accurately transform the social and economic data in the administrative unit into the grid data of the evaluation unit has become a key issue.

In general, the health evaluation index of cultivated land systems should consider both macro-level management control and micro-level implementation feasibility due to the inherent characteristics of heterogeneity and diversity of cultivated land systems, the complexity and availability of multi-source data, and the varying objectives and needs of stakeholders at different levels. Future research should focus on the effects of climate change, annual accumulated temperature, annual precipitation index, ecosystem services, and other factors on the health of cultivated land systems on a macro scale [78,79,80] and on the effects of heavy metal pollution, soil organic carbon, soil erosion, soil organic matter, soil pH, soil nutrient elements (nitrogen, phosphorus, and potassium), soil texture, effective soil depth, and soil microorganisms on the health of cultivated land systems on a micro scale [17,81,82]. In addition, although the grid scale of 5 km × 5 km is utilized as the evaluation unit in this research, the scale effect of agricultural system health assessment must be studied further, with a focus on the impact of grid size on farming system health evaluation results and its spatial-temporal pattern. In order to meet the diverse management needs of people, it is also necessary to improve the accuracy of multi-source data, compare and analyze the health of cultivated land systems at multiple spatial scales, and investigate the effective connection methods of different levels of cultivated land system health in order to provide a reference for regional cultivated land use and planning management.

### 4.2. Policy Enlightenment

Five health status zones of the cultivated land system are divided. To maintain regional food security and support the sustainable growth of agriculture, it is vital to adopt diverse policies for the exploitation and management of cultivated land [69].

(1) The health status in the moderately optimized area has improved significantly. The existing health status should be maintained and controlled with the goal of increasing production, income, and green health, improve the irrigation convenience, accessibility, and fertility of cultivated land, gradually reduce the use of chemical fertilizers and pesticides, and improve the green production level of cultivated land [78,83].
(2) The collaborative optimization zone can rely on its inherent advantages of location and good cultivated land resources to vigorously develop the integration of rural primary, secondary, and tertiary industries, and take characteristic leisure agriculture and ecological agriculture as the leading industries to play a demonstration role in the utilization of cultivated land in other types of areas; we will strengthen the construction and management of high-standard farmland to ensure the safety and stability of the farmland system.
(3) The health status in the potential promotion area is relatively stable. For the local areas with general or poor health status, non-agricultural occupation of cultivated land is strictly prohibited. At the same time, the internal advantages of the area are explored, and ecological agriculture, organic agriculture, and green agriculture are vigorously developed to gradually optimize the regional layout of agricultural planting and agricultural production structure.
(4) The key upgrading area is highly disturbed by human activities, the degree of fragmentation of cultivated land in the area is high, and the stability of cultivated land production is general. The division needs to increase the investment in ecological environment protection of cultivated land and improve the level of ecological restoration and environmental governance; we will strengthen land use control and comprehensive management of multi-functional land, strengthen agricultural pollution control and supervision, and improve the level of carbon sequestration and water conservation functions of cultivated land, as well as optimize the input structure of cultivated land production factors and enhance the scale efficiency and competitive advantage of cultivated land utilization [84].
(5) The priority promotion region is characterized by excessive fragmentation of cultivated land, low usage efficiency of cultivated land, and poor cultivated land system health. The division needs to innovate the mode of cultivated land production and management and change the mode of agricultural development, strengthen the restraint of the “red line” of cultivated land, reduce the impact of urbanization and industrialization on the ecological regulation and landscape carrying capacity of cultivated land, strengthen the monitoring of the health level of the cultivated land system, and combine the soil improvement project with the comprehensive treatment project to build a healthy and reasonable field road network system and realize the efficient and sustainable utilization of cultivated land resources [85].

## 5. Conclusions

This paper constructs, on the basis of the PSR-VOR framework, a health evaluation system for the cultivated land system, which is used to analyze the spatial and temporal pattern of the health of the cultivated land system in the two lake plains and to demarcate different regulation zones for the health of the cultivated land system. The health index of the cultivated land system has fluctuated downward during the past two decades. The amount of cultivated land that is in poor health is fast expanding, despite the fact that the health state of the cultivated land system is typically good. In space, the distribution of the health level of the cultivated land system decreases steadily from the northeast to the southwest and southeast. Moreover, the cultivated land system health index has a high spatial positive autocorrelation, its geographical agglomeration degree exhibits a changing tendency of first growing, then dropping, and then increasing, and spatial heterogeneity is steadily increasing. The distribution of the high-value cluster area and the low-value cluster area of the cultivated land health index shows no discernible change, with high values in the north and low values in the south, decreasing from the center to the periphery. There are considerable disparities in the direction of change in the health status of the cultivated land system throughout different time periods, and the degree of improvement in the cultivated land system’s health status in the northern area is clearly superior to that in the southern region. Optimizing and controlling the health state of the cultivated land system from moderately optimized to priority areas is becoming increasingly urgent. The future management and conservation of cultivated land resources should be diversified based on local characteristics, which is crucial for achieving the sustainable development of regional agriculture.

## Figures and Tables

**Table 1:** Equivalent value of farmland ecosystem services per unit area in the two lake plains (yuan·hm^2^·a^−1^).

Main Class	Sub Class	Ecosystem Service Value Equivalent
Supply service	Grain production	2581.143
	Raw material production	1006.646
Regulation service	Gas regulation	1858.423
	Climate regulation	2503.709
	Hydrological regulation	1987.480
	Waste disposal	3587.789
Support services	Soil conservation	3794.280
	Biodiversity maintenance	2632.766
Cultural Services	Aesthetic landscape	438.794

**Table 2:** Health evaluation index system in the two lake plains.

Target Layer	Criterion Layer	Factor Layer	Indicator Layer	Indicator Attribute	Initial Weight	Comprehensive Weight
Health of cultivated land utilization system (A_1_)	System pressure (B_1_)	Population growth pressure (C_1_)	Population density (D_1_)	−	0.0057	0.0130
			Urbanization rate (D_2_)	−	0.0453	−
		Economic driving force (C_2_)	GDP per capita (D_3_)	−	0.0449	−
			Economic density (D_4_)	−	0.0032	0.0302
		Cultivated land use intensity (C_3_)	Land reclamation rate (D_5_)	−	0.004	0.0338
			Pesticide application intensity (D_6_)	−	0.0029	0.0385
			Application intensity of agricultural chemical fertilizer (D_7_)	−	0.0033	0.0401
			Agricultural plastic film amount (D_8_)	−	0.0029	−
	System status (B_2_)	Vitality (C_4_)	Cultivated land productivity (D_9_)	+	0.049	0.0520
			NDVI (D_10_)	+	0.0044	0.0336
		Ecological structure index (C_5_)	Landscape fragmentation index (D_11_)	−	0.0499	−
			Biological abundance (D_12_)	+	0.0043	0.0335
		Economic structure index (C_6_)	Total agricultural output value per capita (D_13_)	+	0.0484	−
			Proportion of total agricultural output value (D_14_)	+	0.0616	0.0753
		Social structure index (C_7_)	Proportion of agricultural employees (D_15_)	+	0.0268	0.0396
			Rural population carrying capacity per unit area (D_16_)	−	0.0486	−
		Restoring force (C_8_)	Per capita cultivated land area (D_17_)	+	0.0243	0.0395
			Ecological elasticity (D_18_)	+	0.0048	−
	System response (B_3_)	Socio economic response (C_9_)	Grain per capita (D_19_)	+	0.0248	0.0389
			Per capita net income of farmers (D_20_)	+	0.0248	0.0286
			Agricultural mechanization level (D_21_)	+	0.0372	0.0577
			Agricultural power intensity (D_22_)	+	0.0214	0.0285
			Irrigation assurance rate (D_23_)	+	0.0106	−
		Environmental ecological response (C_10_)	Climate regulation function (D_24_)	+	0.0903	0.1044
			Waste treatment function (D_25_)	+	0.0903	0.1044
			Soil conservation function (D_26_)	+	0.0903	0.1044
			Maintain biodiversity function (D_27_)	+	0.0903	0.1044

Note: “−” indicates a negative indicator; “+” indicates a positive indicator.

**Table 3:** Type, sources and spatial resolution of data used in this study.

Type	Source	Spatial Resolution
Land cover data	Resource and Environment Science and Data Center, CAS	30 m × 30 m
Landsat 8	Geospatial Data Cloud	30 m × 30 m
Agricultural production data	Statistical Yearbook of Hubei and Hunan Provinces	County scale
NDVI	National Earth System Science Data Sharing Platform in China	500 m × 500 m
NPP	National Earth System Science Data Sharing Platform in China	1 km × 1 km
DEM	Geospatial Data Cloud	30 m × 30 m
Soil data	Soil Science Database of China	1:1,000,000
Administrative divisions and road data	National basic geographic information center of China	1:250,000

**Table 4:** Changes in cultivated land area of different levels in the two lake plains.

Year	Statistical Content	Class V	Class IV	Class III	Class II	Class I
2000–2005	Variation (hm^2^)	22,886.55	42,658.65	58,448.79	−15,957.00	−168,544.80
	Change rate (%)	80.33%	21.19%	11.20%	−1.28%	−9.56%
2005–2010	Variation (hm^2^)	9311.76	24,705.00	152,186.85	384,302.07	−593,912.25
	Change rate (%)	18.12%	10.12%	26.22%	31.13%	−37.24%
2010–2015	Variation (hm^2^)	32,720.35	136,431.78	180,396.46	192,975.91	−647,476.96
	Change rate (%)	53.92%	50.77%	24.62%	11.92%	−64.68%
2015–2019	Variation (hm^2^)	10,567.67	29,787.51	37,202.75	−227,291.56	−171,282.38
	Change rate (%)	11.31%	7.35%	4.07%	−12.54%	−48.45%
2000–2019	Variation (hm^2^)	75,486.33	233,582.94	428,234.85	334,029.42	−1,581,216.39
	Change rate (%)	264.96%	116.01%	82.03%	26.71%	−89.67%

**Table 5:** Statistics of cultivated land area of different levels of cultivated land system health.

Year	Statistical Content	Class V	Class IV	Class III	Class II	Class I
2000	Area (hm^2^)	28,489.32	201,355.47	522,077.4	1,250,622.18	1,763,452.62
	Proportion (%)	0.76%	5.35%	13.86%	33.21%	46.83%
2005	Area (hm^2^)	51,375.87	244,014.12	580,526.19	1,234,665.18	1,594,907.82
	Proportion (%)	1.39%	6.59%	15.67%	33.32%	43.04%
2010	Area (hm^2^)	60,687.63	268,719.12	732,713.04	1,618,967.25	1,000,995.57
	Proportion (%)	1.65%	7.30%	19.90%	43.97%	27.19%
2015	Area (hm^2^)	93,407.98	405,150.90	913,109.50	1,811,943.16	353,518.61
	Proportion (%)	2.61%	11.33%	25.53%	50.65%	9.88%
2019	Area (hm^2^)	103,975.65	434,938.41	950,312.25	1,584,651.6	182,236.23
	Proportion (%)	3.19%	13.36%	29.19%	48.67%	5.60%

**Table 6:** Spatial transformation rules of cultivated land system health condition.

Change Level		2019				
		Best Health (I)	Better Health (II)	Medium Health (III)	Poor Health (IV)	Poorer Health (V)
2000	Best Health (I)	11	12	13	14	15
	Better Health (II)	21	22	23	24	25
	Medium Health (III)	31	32	33	34	35
	Poor Health (IV)	41	42	43	44	45
	Poorer Health (V)	51	52	53	54	55

Note: I stands for best health condition, II stands for better health condition, III stands for medium health condition, IV stands for poor health condition, and V stands for poorer health condition. If there is no box in this table, it means that this type of change has not occurred.

**Table 7:** Zoning methods and characteristics of health status.

Health Condition Area	Category	Cultivated Land Area (hm^2^)	Main Features
Moderate optimization area	31/41/42/52/53	36,925.57	The health condition has improved significantly, and it has been upgraded by two grades as a whole.
Collaborative optimization area	21/32/43/54	134,011.15	The health condition has been slightly improved, and the overall level has been raised by one level.
Potential promotion area	11/22/33/44/55	911,500.41	The health condition is relatively stable, with little overall change.
Key promotion area	12/23/34/45	1,781,765.18	The health condition deteriorated slightly, and the overall situation dropped by one grade.
Priority promotion area	13/14/24/25/35	428,720.23	The health condition deteriorated obviously, and the whole declined by two grades.

## Data Availability

Not applicable.
